# Role of the novel endoribonuclease SLFN14 and its disease-causing mutations in ribosomal degradation

**DOI:** 10.1261/rna.066415.118

**Published:** 2018-07

**Authors:** Sarah J. Fletcher, Vera P. Pisareva, Abdullah O. Khan, Andrew Tcherepanov, Neil V. Morgan, Andrey V. Pisarev

**Affiliations:** 1 Institute of Cardiovascular Sciences, College of Medical and Dental Sciences, University of Birmingham, Birmingham B15 2TT, United Kingdom; 2 Department of Cell Biology, SUNY Downstate Medical Center, Brooklyn, New York 11203, USA; 3 Department of Physiology and Pharmacology, SUNY Downstate Medical Center, Brooklyn, New York 11203, USA

**Keywords:** endoribonuclease, platelet, ribonuclease, RNA degradation, SLFN14, thrombocytopenia

## Abstract

Platelets are anucleate and mostly ribosome-free cells within the bloodstream, derived from megakaryocytes within bone marrow and crucial for cessation of bleeding at sites of injury. Inherited thrombocytopenias are a group of disorders characterized by a low platelet count and are frequently associated with excessive bleeding. *SLFN14* is one of the most recently discovered genes linked to inherited thrombocytopenia where several heterozygous missense mutations in *SLFN14* were identified to cause defective megakaryocyte maturation and platelet dysfunction. Yet, SLFN14 was recently described as a ribosome-associated protein resulting in rRNA and ribosome-bound mRNA degradation in rabbit reticulocytes. To unveil the cellular function of SLFN14 and the link between SLFN14 and thrombocytopenia, we examined SLFN14 (WT/mutants) in in vitro models. Here, we show that all *SLFN14* variants colocalize with ribosomes and mediate rRNA endonucleolytic degradation. Compared to SLFN14 WT, expression of mutants is dramatically reduced as a result of post-translational degradation due to partial misfolding of the protein. Moreover, all SLFN14 variants tend to form oligomers. These findings could explain the dominant negative effect of heterozygous mutation on SLFN14 expression in patients’ platelets. Overall, we suggest that SLFN14 could be involved in ribosome degradation during platelet formation and maturation.

## INTRODUCTION

Inherited thrombocytopenias (ITs) are a group of disorders determined by a relative decrease of platelet count resulting from genetic heterogeneity (Nurden and Nurden 2007). ITs are usually asymptomatic, but some individuals may experience excessive bleeding ranging from mild to severe. Over 30 genes are shown to be involved in ITs (Favier and Raslova 2015; Levin et al. 2015; Johnson et al. 2016a,b; Pecci 2016). One of these, *SLFN14*, was discovered only very recently where Fletcher et al. (2015) identified three heterozygous missense mutations in affected family members from three unrelated families, predicted to encode substitutions K218E, K219N, and V220D within an AAA domain of SLFN14. Patients revealed moderate IT with severe bleeding history and platelet ATP secretion defects. Importantly, SLFN14 expression is dramatically reduced in patients’ platelets (up to 80%) compared to healthy controls and in transfected cells (up to 95%), suggesting a dominant negative effect of mutants on the synthesis or stability of the wild-type (WT) form (Fletcher et al. 2015). Shortly after this seminal study, Marconi et al. (2016) reported the fourth heterozygous missense mutation in *SLFN14* associated with IT. Notably, the affected residue R223W in the AAA domain of SLFN14 is located nearby to previously reported mutations. This novel mutation was shown to mediate reduced proplatelet formation and decreased megakaryocyte maturation in patient derived megakaryocytes. Consistent with above mentioned data, SLFN14 expression was below 50% despite the heterozygous nature of the mutation (Marconi et al. 2016). This finding supports the idea of dominant negative effect of mutant forms on the SLFN14 WT expression (Fletcher et al. 2015; Marconi et al. 2016). However, due to the limited knowledge of the function of SLFN14, more detailed characterization of *SLFN14* and its role in platelet biogenesis is critically important.

Alongside these genetic studies, Pisareva et al. (2015) demonstrated the endoribonucleolytic activity of purified SLFN14 in biochemical experiments and suggested the role of this protein in translation control. More specifically, it was shown that SLFN14 associates with ribosomes and ribosomal subunits, and cleaves RNA, but preferably rRNA and ribosome-bound mRNA, in a Mg^2+^-dependent and NTP-independent manner. This leads to the degradation of ribosomal subunits (Pisareva et al. 2015). More recently a more global approach (Mills et al. 2016) described a study of a ribosomal rescue pathway which involves both erythroid cells and platelets and the many proteins involved in this process such as proteins like SLFN14.

Based on the presence of characteristic slfn signature motifs, SLFN14 belongs to the Schlafen protein family, limited to mammals and encoded by six *SLFN* genes in humans (Geserick et al. 2004; Brady et al. 2005). All family members comprise a conserved amino terminus containing a putative AAA domain implicated in ATP binding, but only longer forms of *SLFN* genes (including *SLFN14*) possess a carboxy-terminal extension with motifs, which are specific for superfamily I DNA/RNA helicases (Geserick et al. 2004). SLFN proteins are involved in T-cell development (Schwarz et al. 1998; Geserick et al. 2004; Berger et al. 2010), differentiation (Patel et al. 2009), and immune response (Li et al. 2012), but their exact cellular functions still remain elusive.

To get insights into the fundamental role of SLFN14, we aimed to assay endoribonuclease activity, intracellular distribution, and stability of WT and IT-related missense mutation forms of protein in human cells. We found that SLFN14 colocalizes with ribosomes and causes the endoribonucleolytic degradation of rRNA in cells. Mutations do not affect all tested activities of the protein, but dramatically reduce mutants’ stability at post-translational level and down-regulate the coexpression of WT form. In light of our data, implications for the fundamental role of SLFN14 are discussed.

## RESULTS

### SLFN14 WT and IT-related missense mutation variants reveal the same subcellular distribution, colocalize with 5.8S rRNA, and cause ribosome degradation in Dami cells

**FIGURE 1.**
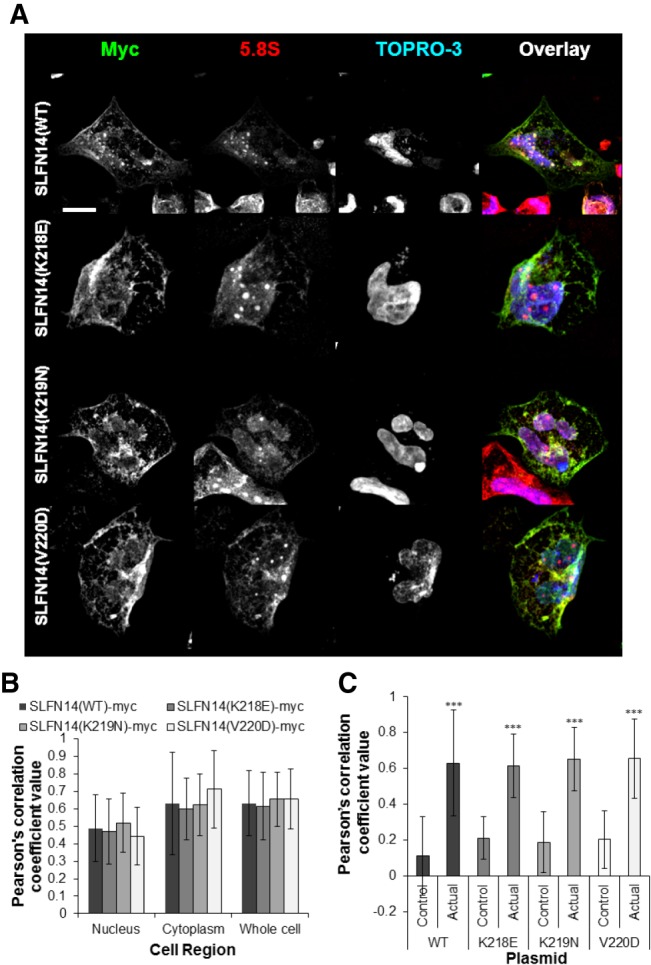
Significant colocalization is observed between SLFN14 (WT/mut)-myc and 5.8S rRNA in differentiated Dami cells. There is no alteration in subcellular distribution between SLFN14 (WT)-myc and SLFN14 (mut)-myc distribution or colocalization with 5.8S rRNA. (*A*) Transiently transfected differentiated Dami cells expressing SLFN14(WT/mut)-myc for 48 h were probed with rabbit anti-myc and mouse anti-5.8S primary antibodies followed by incubation with anti-rabbit AlexaFluor488, anti-mouse AlexaFluor568 secondary antibodies and TO-PRO-3 Iodide nuclear stain. A representative image is shown from three independent experiments, scale bar denotes 15 µm. (*B*) Pearson's correlation coefficient data demonstrating no change in colocalization or subcellular-distribution between 5.8S rRNA and SLFN14 (WT)-myc or SLFN14 (mut)-myc in comparison to control areas. *n* = at least 40 cells analyzed from three independent experiments. (*C*) Pearson's correlation coefficient data demonstrating a significant increase in colocalization between 5.8S rRNA and SLFN14 (WT/mut)-myc staining in comparison to control areas. (***) *P* ≤ 0.001 colocalization between 5.8S rRNA and SLFN14 (WT/mut)-myc and control area. Error bars ± SD.

To date, data on SLFN14 activity are limited by describing the protein as an endoribonuclease in a rabbit reticulocyte lysate and in a reconstituted in vitro mammalian translation system (Pisareva et al. 2015). To expand our knowledge on the function of this protein, we aimed to analyze SLFN14 in transfected cell lines. Therefore, we used myc-tagged human SLFN14 WT (SLFN14(WT)-myc) and three previously reported missense mutation variants K218E, K219N, and V220D (SLFN14(K218E)-myc, SLFN14(K219N)-myc, and SLFN14(V220D)-myc, respectively) that were cloned into the mammalian expression vector for transient expression (Fletcher et al. 2015). Dami cells, a human megakaryocytic leukemia cell line, were selected as a host cell line. Dami cells were differentiated into megakaryocyte like cells using Phorbol 12-myristate 13-acetate (PMA) then transiently transfected with SLFN14 (WT/mut)-myc constructs for 48 h and were subsequently analyzed by immunostaining. Expression of SLFN14 (WT)-myc revealed a diffuse cytoplasmic and nuclear localization with some punctate structures observed (Fig. 1A). None of the mutations affected the subcellular distribution and staining pattern of the protein (Fig. 1A,B). Such a pattern may indicate the colocalization of SLFN14 with nonuniform dispersed components of the cell. It was previously shown that SLFN14 strongly binds to purified ribosomes in the sucrose density gradient (SDG) centrifugation experiment (Pisareva et al. 2015). Therefore, we suggested that ribosomes could be a binding partner for SLFN14 in the cell. Immunostaining experiments with the antibodies against 5.8S rRNA revealed that SLFN14 (WT)-myc significantly colocalizes with ribosomes, and the presence of mutations do not influence this colocalization (Fig. 1A–C). SLFN14 was reported to possess the ribosomal binding site within 179 amino acids in the central part of the protein beside the AAA domain to the carboxyl terminus (Pisareva et al. 2015). Therefore, we proposed that SLFN14 should bind directly to the ribosomes in cells.

**FIGURE 2.**
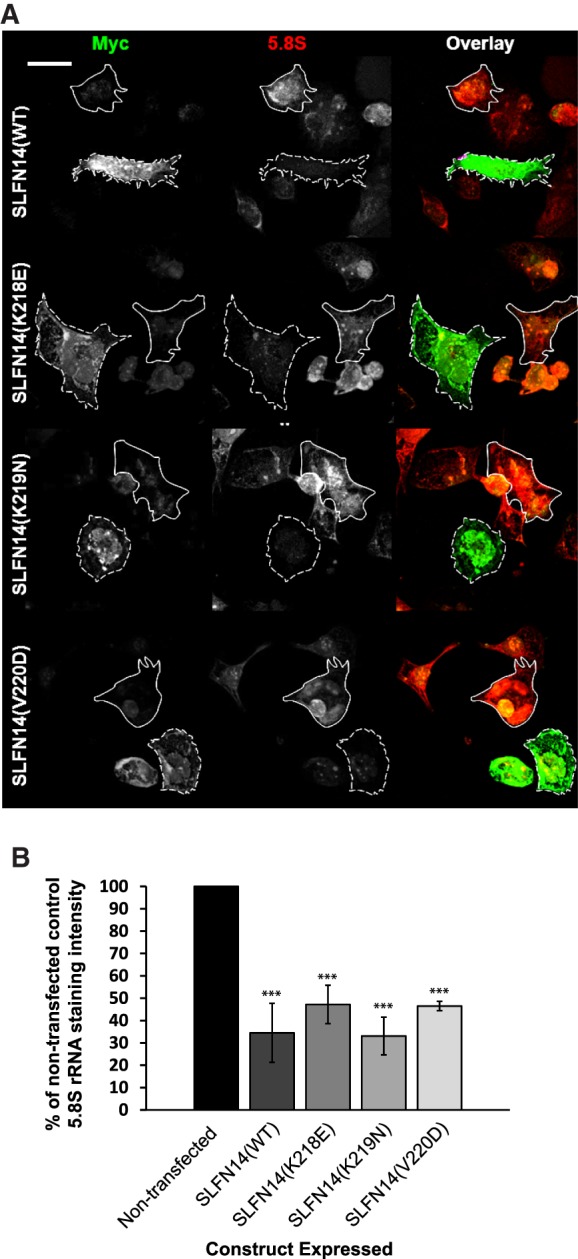
Reduced intensity of 5.8S rRNA staining in differentiated Dami cells expressing wild-type/mutant SLFN14 constructs. (*A*) Transiently transfected Dami cells expressing SLFN14(WT/mut)-myc for 48 h were probed with rabbit anti-myc and mouse anti-5.8S primary antibodies followed by incubation with anti-rabbit AlexaFluor488 and anti-mouse AlexaFluor568 secondary antibodies, respectively. The dashed white line outlines cells expressing SLFN14 (WT/mut)-myc, the solid line represents the outline of cells which were not transfected. A representative image is shown from three independent experiments. Scale bar denotes 15 µm. (*B*) Average intensity measurements from the entire cell area were quantified from images represented in *A*. *n* = at least 40 cells analyzed from three independent experiments. (***) *P* ≤ 0.001 when compared to nontransfected cells. Error bars ± SD.

Rabbit SLFN14 was previously shown to cause rRNA cleavage and ribosome degradation in a rabbit reticulocyte lysate and in a reconstituted in vitro mammalian translation system (Pisareva et al. 2015). Notably, SLFN14 is highly homologous among all mammalian species. To test whether SLFN14 could provide ribosome degradation in the intact cells of human origin, we estimated 5.8S rRNA content in transiently transfected Dami cells expressing SLFN14 (WT/mut)-myc for 48 h by immunostaining of 5.8S rRNA. For all SLFN14 variants, we detected ∼50% to 70% statistically significant reduction of 5.8S rRNA content (Fig. 2). Based on staining intensity values (Fig. 2B), we cannot state that mutations compromise the ribosome degradation activity of SLFN14. However, taking into account previous biochemical data (Pisareva et al. 2015), we suggest the more direct rather than auxiliary role of SLFN14 in the ribosome degradation.

In conclusion, SLFN14 binds to the ribosomes mediating ribosome degradation in megakaryocyte-like cells, and IT-related missense mutations do not compromise these cellular activities of protein assuming that SLFN14 dysfunction takes place at a different molecular level.

### Overexpressed SLFN14 WT and mutants associate with ribosomes and individual ribosomal subunits causing the endoribonucleolytic cleavage of rRNA in HEK293T cells

**FIGURE 3.**
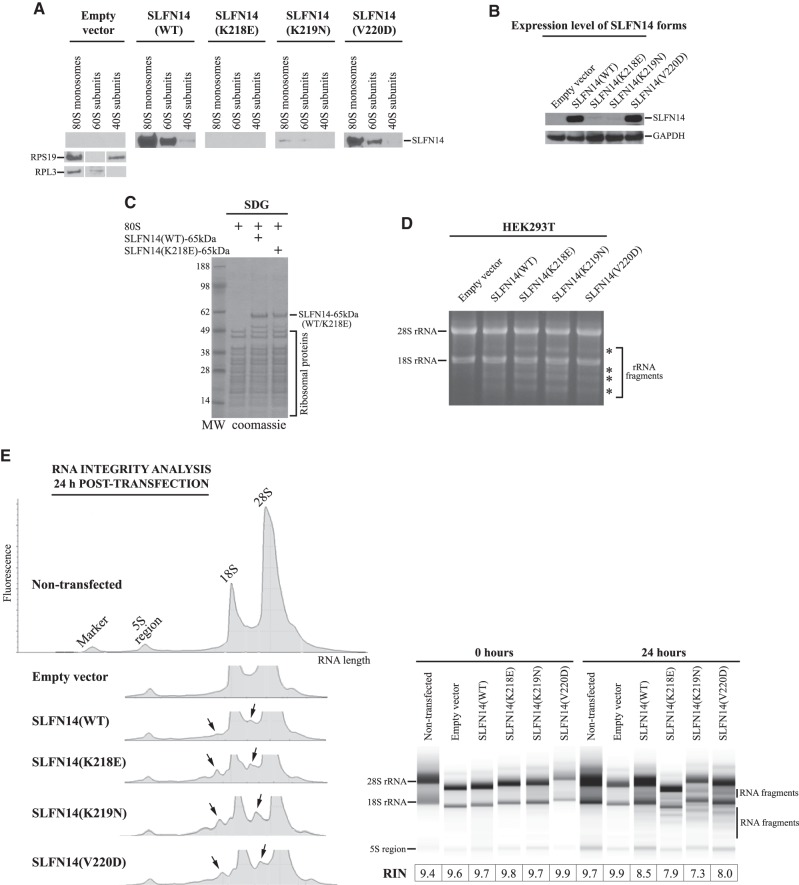
Association of SLFN14 (WT/mut)-myc with ribosomes and ribosomal subunits resulting in rRNA endonucleolytic degradation in HEK293T cells. (*A*) Binding of SLFN14 (WT/mut)-myc to 80S monosomes, 60S, and 40S ribosomal subunits obtained after SDG centrifugation of HEK293T cell lysates with overexpressed one of SLFN14 (WT/mut)-myc forms. Assignment of 80S monosomes, 40S, and 60S subunits in gradient fractions was based on immunoblotting with anti-RPS19 and anti-RPL3 antibodies as exemplified by the EV control. Ribosomal fractions were concentrated and analyzed by immunoblotting with anti-SLFN14 antibodies. (*B*) Expression levels of SLFN14 (WT/mut)-myc in HEK293T cells assayed by immunoblotting with anti-SLFN14 and anti-GAPDH (control) antibodies. (*C*) Binding of recombinant SLFN14 (WT)-65 kDa and SLFN14 (K218E)-65 kDa to assembled 80S ribosomes assayed by SDG centrifugation and Coomassie staining. (*D*) rRNA degradation in SLFN14(WT/mut)-overexpressed HEK293T cells assayed by denaturing agarose/formaldehyde gel electrophoresis (*n* = 3 independent experiments). The asterisks indicate the main bands of rRNA fragments. Positions of the 28S rRNA and 18S rRNA are shown. (*E*) Integrity analysis of rRNA. (*Left* panel) Representative electrophoretic profiles of total RNA obtained using the Agilent 2200 TapeStation system from SLFN14(WT/mut)-overexpressed HEK293T cells. Arrows indicate the appearance of rRNA degradation fragments. (*Right* panel) representative data output gel-like images. The average RIN scores from three independent experiments are shown.

To evaluate whether the discovered SLFN14 activities are related to a megakaryocyte-specific cell line, we also assayed the protein in HEK293T cells, a human embryonic kidney cell line. To analyze the ribosomal binding of SLFN14, transiently transfected HEK293T cells expressing SLFN14(WT/mut)-myc or harbouring empty vector (EV) for 48 h were collected and lysed, and cell lysates were subjected to SDG centrifugation in order to obtain 80S-, 60S-, and 40S-containing fractions. Notably, we did not observe the significant difference in polysome profiles upon SLFN14(WT/mutants) expression compared to EV control. All SLFN14 forms except SLFN14 (K218E)-myc bind predominantly to 80S ribosomes and 60S ribosomal subunits (Fig. 3A). The weak signals of SLFN14 (K219N)-myc and the absence of SLFN14 (K218E)-myc in ribosomal peaks correlate with the low expression level of proteins (Fig. 3B). It was previously shown that the expression level of SLFN14 (K218E)-myc in HEK293T cells is the lowest among all the described mutants (Fletcher et al. 2015). Therefore, we hypothesized that the absence of SLFN14 (K218E)-myc in ribosomal fractions is a result of the low content and/or continuous degradation during cell lysate manipulation rather than of the compromised ribosomal binding activity. To test our hypothesis, we utilized a previously described *Escherichia coli* expression vector for a 65 kDa carboxy-terminally truncated His-tagged form of human SLFN14 (SLFN14-65 kDa) (Pisareva et al. 2015). This was the longest form of SLFN14, which was available in a soluble state after expression, whereas all longer forms completely precipitated (Pisareva et al. 2015). We introduced the corresponding mutation into SLFN14-65 kDa to obtain K218E mutant expression vector [SLFN14 (K218E)-65 kDa]. Due to limited solubility, SLFN14-65 kDa or SLFN14 (K218E)-65 kDa proteins in the form of eluates after Ni-NTA resin were mixed with 80S ribosomes reconstituted from purified 40S and 60S ribosomal subunits. The reaction mixture was subjected to the centrifugation through SDG to separate 80S ribosomes from unbound components, and the 80S ribosomal peak was assayed by denatured PAGE and Coomassie staining. Both SLFN14-65 kDa and SLFN14 (K218E)-65 kDa were found associated with 80S ribosomes (Fig. 3C). Therefore, none of the SLFN14 mutations affect the ribosomal binding activity of SLFN14 in different cell lines.

In the immunostaining experiment, all SLFN14 forms reduced the intensity of 5.8S rRNA staining in Dami transfected cells that could be a result of ribosome degradation. In our next experiment, we raised several key questions. Could the rRNA degradation process be involved in the elimination of ribosomes? Is the degradation activity of SLFN14 cell type-specific? How does the rRNA pattern look after the overexpression of SLFN14? To answer these questions, transiently transfected HEK293T cells expressing SLFN14(WT/mut)-myc or harbouring EV for 24 h were collected, total RNA was isolated, and the equal amounts of total RNA from each sample were assayed by denaturing agarose/formaldehyde gel electrophoresis. In contrast to EV, overexpression of any SLFN14 form resulted in the characteristic pattern of rRNA endoribonuclealytic degradation (Fig. 3D).

To support our data, we analyzed RNA samples derived from either nontransfected HEK293T cells or cells transfected with the empty myc-vector/SLFN14(WT/Mut)-myc for the RNA integrity using the Agilent 2200 TapeStation system. The Agilent system generates an electropherogram profile, gel-like image, and RNA integrity number (RIN) providing the information about RNA quality. Our study demonstrates excellent RIN scores for nontransfected and EV-transfected samples, and lower RIN scores, reflecting partial degradation of rRNA, for SLFN14(WT/Mut)-transfected samples (Fig. 3E) indicating RNA degradation seen in Figure 3D is due to the activity of SLFN14(WT/Mut) overexpression. In summary, the endoribonucleolytic activity of SLFN14 should relate to the observed ribosome degradation in transfected cells. Notably, IT-related missense mutations do not affect rRNA cleavage pattern pointing to that SLFN14 dysfunction in platelet biogenesis cannot be explained by the compromised endoribonucleolytic activity of protein.

### *SLFN14* missense mutations cause the low expression of mutants as a result of post-translational degradation due to partial misfolding and implicate SLFN14 WT into the degradation through the formation of oligomeric forms

**FIGURE 4.**
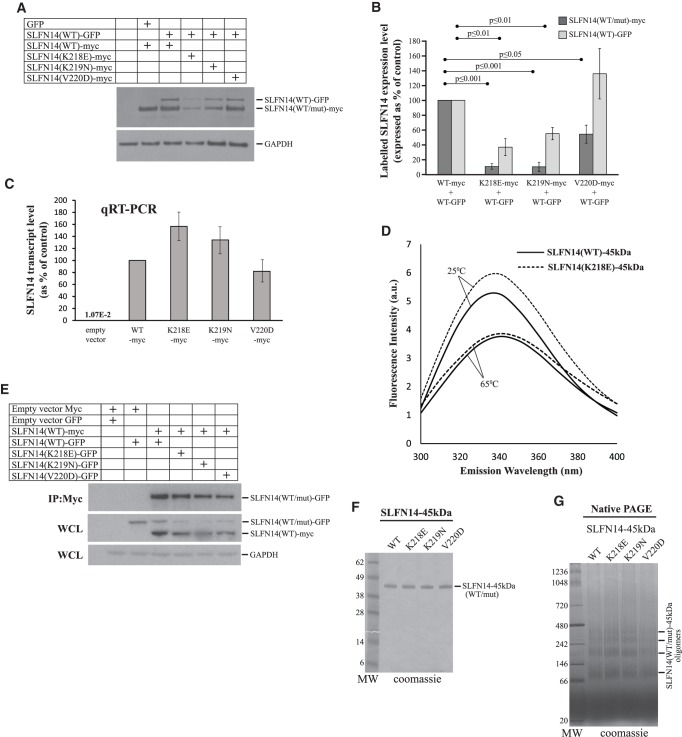
Role of *SLFN14* missense mutations in protein expression. (*A*) Immunoblotting image showing levels of SLFN14 (WT/mut)-myc and SLFN14 (WT)-GFP in HEK293T cells transiently expressing the above constructs. The blot was probed with anti-SLFN14 and anti-GAPDH primary antibodies followed by incubation with anti-rabbit HRP. (*B*) Quantification of SLFN14 (WT/mut)-myc and SLFN14 (WT)-GFP protein expression from immunoblotting analysis of *n* = 3 lysate samples per condition from three independent experiments. All values are mean ± SD. (*C*) Quantification of relative SLFN14 (WT/mut)-myc transcript level normalized to GAPDH control transcript from qRT-PCR test data on *n* = 6 independent sets of lysate samples. All values are mean ± SD. (*D*) Fluorescence emission spectra of SLFN14 (WT)-45 kDa and SLFN14 (K218E)-45 kDa proteins collected at the same protein concentration at two different temperatures: 25°C and 65°C (*n* = 3 independent experiments). (*E*) Coimmunoprecipitation (co-IP) analysis in HEK293T cells to assess both wild-type and mutant GFP labeled SLFN14 with SLFN14(WT)-myc to look at formation of hetero-oligomers between wild-type and mutant SLFN14. (*F*) Purified recombinant SLFN14-45 kDa WT and mutants resolved by SDS-PAGE. (*G*) Oligomerization capacity of SLFN14-45 kDa WT and mutants assayed by native PAGE. Positions of different oligomeric forms are shown.

One of the interesting reported findings is that SLFN14-related IT patients displayed 65%–80% reduction in SLFN14 protein level despite the heterozygosity, assuming that the mutant allele influences the synthesis and/or stability of both mutant and WT proteins (Fletcher et al. 2015; Marconi et al. 2016). This effect was confirmed in overexpression studies in transfected cells (Fletcher et al. 2015). Therefore, in our next set of experiments, we aimed to determine the stage, at which mutant expression could be affected, and to understand how mutants could influence the SLFN14 WT protein level. For that, we used above mentioned SLFN14 (WT/mut)-myc vectors and a newly constructed SLFN14 (WT)-GFP mammalian expression vector containing the human *SLFN14* coding region with a GFP tag. Transiently transfected HEK293T cells coexpressing SLFN14 (WT)-GFP and either the SLFN14 (WT/mut)-myc vectors for 48 h were analyzed by immunoblotting. First, consistently with published data (Fletcher et al. 2015), we found that SLFN14 (K218E)-myc, SLFN14 (K219N)-myc, and SLFN14 (V220D)-myc expression was reduced to 10%, 10%, and 54% of SLFN14(WT)-myc expression, respectively (Fig. 4A,B). Second, SLFN14(WT)-GFP protein level dropped to 37% and 54% upon coexpression of that with SLFN14(K218E)-myc and SLFN14(K219N)-myc, respectively, compared to coexpression with SLFN14(WT)-myc control (Fig. 4A,B). These data correlate with the proposed dominant negative effect of SLFN14 mutants. The detected elevation of SLFN14 (WT)-GFP protein level upon coexpression with SLFN14 (V220D)-myc compared to that with the SLFN14 (WT)-myc control was not statistically significant and we therefore suggest that this mutant behaves in a different way (Fig. 4A,B).

The regulation of expression of SLFN14 mutants should take place at either the transcriptional or post-translational level. To evaluate the effect of mutations on the RNA level, we used qRT-PCR. We isolated total RNA from HEK293T cells expressing one of SLFN14 (WT/mut)-myc variants, and assayed samples for expression of *SLFN14* and *GAPDH* control transcripts. The difference in a normalized transcript concentration between *SLFN14* WT and each mutant form did not exceed 1.6-fold (Fig. 4C). Importantly, the endogenous *SLFN14* transcript was not detected (Fig. 4C). Taking into account a strong reduction of SLFN14 (K218E)-myc and SLFN14 (K219N)-myc expression, we conclude that the regulation of expression should occur at a post-translational rather than a transcriptional stage, at least for these two mutants. We then hypothesized that missense mutations could affect protein folding. To test this hypothesis, we compared folding between the SLFN14 WT and K218E form, which revealed the most dramatic effect on its own expression and WT form coexpression in our experiments. Tertiary structure of protein can be analyzed using a fluorescence spectroscopy technique based on intrinsic protein fluorescence. Two amino acids, Trp and Tyr, are experimentally used to obtain a strong fluorescent signal. The emission energy of these residues is highly sensitive to the polarity of the environment. In the native folded conformation, Trp and Tyr are generally hidden in the hydrophobic core of the protein giving a high intensity fluorescence signal. In contrast, a hydrophilic environment results in a low intensity fluorescence signal. The protein sample is excited at 280-nm wavelength, and fluorescence spectrum is collected in a 300- to 400-nm wavelength range. For the experiment, we used the already described *E. coli* expression vector for 45 kDa carboxy-terminally truncated His-tagged form of human SLFN14 (SLFN14-45 kDa) (Pisareva et al. 2015). Compared to SLFN14-65 kDa and longer protein forms, SLFN14-45 kDa could be purified in a large amount with high homogeneity (Pisareva et al. 2015). We inserted the corresponding mutation into SLFN14-45 kDa to obtain K218E mutant expression vector (SLFN14 (K218E)-45 kDa). Analysis of SLFN14-45 kDa and SLFN14(K218E)-45 kDa fluorescence spectra revealed the difference in the maximum of emission energy at 340 nm wavelength indicating different conformations of proteins and confirming our hypothesis (Fig. 4D). In the control experiment, after incubation of proteins at 65°C resulting in their denaturation, maximum intensities of emission matched (Fig. 4D). In conclusion, we state that *SLFN14* missense mutations lead to post-translational degradation of mutants as a result of partial protein misfolding.

Taking into account a post-translational regulation of *SLFN14* expression, we suggested that mutants could involve WT protein with degradation through the formation of heterogeneous oligomeric forms. Indeed, AAA proteins form oligomeric assemblies, mostly homo-hexamers, which are critical for their activities (Ogura and Wilkinson 2001). To evaluate the SLFN14 tendency to oligomerization we performed coimmunoprecipitation (co-IP) studies and assayed the mobility of protein in native PAGE. For co-IP studies HEK293T cells coexpressing SLFN14(WT)-myc and SLFN14(WT/mut)-GFP for 48 h were lysed, SLFN14(WT)-myc was pulled down using mouse anti-myc antibodies, and IP lysates were analyzed by immunoblotting. Figure 4E demonstrates co-IP of both wild-type and mutant GFP labeled SLFN14 with SLFN14(WT)-myc suggesting the formation of hetero-oligomers between wild-type and mutant SLFN14. Furthermore, we used the above mentioned SLFN14-45 kDa and SLFN14 (K218E)-45 kDa proteins as well as newly constructed, *E. coli* expressed and purified SLFN14 (K219N)-45 kDa and SLFN14 (V220D)-45 kDa mutants (Fig. 4F) to assay the mobility of protein in native PAGE. As a result, SLFN14 WT migrated in a native PAGE in the form of homo-oligomers of different orders, and none of the mutations affected the protein pattern (Fig. 4G). Therefore, we suggest that our findings underlie the mechanism of *SLFN14* expression regulation in transfected cells.

## DISCUSSION

*SLFN14* is one of the most recently discovered genes known to cause IT. Four missense mutations of *SLFN14* are identified to date, linked to dysregulated platelet maturation and platelet dysfunction, resulting in disproportionate bleeding in affected patients. Schlafen family members are poorly studied and their functions are not completely understood making the cellular role of SLFN14 hard to predict. The only functional characterization report describes SLFN14 as an endoribonuclease in a rabbit reticulocyte lysate. To advance our knowledge on SLFN14, we characterized the protein in different transfected cell lines.

Consistent with published data, we detected a diffuse immunostaining pattern for overexpressed SLFN14 WT throughout the nucleus and cytoplasm with some punctate structures in Dami cells. Immunostaining assay also revealed that the nonuniform distribution of SLFN14 WT is a result of colocalization with 5.8S rRNA indicating the ribosome as a binding partner for the protein in the cell. Taking into account the reported biochemical data, we assayed the rRNA degradation capacity of protein in question, and found that SLFN14 WT overexpression leads to rRNA endoribonucleolytic cleavage and degradation in Dami and HEK293T cells, which represent megakaryocyte-related and unrelated cell lines, respectively. Therefore, SLFN14 is a bona fide mammalian endoribonuclease. Importantly, only few endoribonucleases have been described so far. That is because it is hard to predict the endoribonucleolytic activity of protein based on its primary sequence due to a high variety in the organization of the active center and, thus, a wide structural diversity of this class of enzymes. Notably, IT-related missense mutations K218E, K219N, and V220D do not affect the distribution, 5.8S rRNA colocalization, and endoribonucleolytic activity of SLFN14 in the cell. This data implies that protein dysfunction in platelet biogenesis takes place at a different cellular level.

Here we show that all SLFN14 forms bind to ribosomes in cells. Notably, as follows from the experiment in the binary system with the recombinant protein, ribosomal association of SLFN14 is direct and not mediated by cofactors. This is the first reported case of direct ribosomal association of endoribonuclease within the cell.

It was reported that *SLFN14* missense mutations cause the decreased protein expression in transfected cells and down-regulate the expression of SLFN14 WT form in patients. Consistently, in our study, the protein level dropped dramatically for K218E and K219N mutants and moderately for V220D mutant in HEK293T cells. Moreover, GFP-tagged SLFN14 (WT) protein levels reduced by 3 and 2 times upon coexpression with K218E and K219N mutants, respectively, compared to coexpression with WT control. qRT-PCR data on the SLFN14 transcript levels displayed that regulation of expression takes place at a post-translational rather than a transcriptional stage. We hypothesized that the missense mutations could cause partial misfolding of protein. Indeed, to prevent the potentially hazardous effect, the cell uses several degradation pathways to destroy improperly folded proteins (Nedelsky et al. 2008; Smith et al. 2011; Varshavsky 2012). Interestingly, protein misfolding is shown to underlie hundreds of diseases (Valastyan and Lindquist 2014). Fluorescence spectroscopy demonstrated different tertiary structures of SLFN14 WT and K218E supporting our suggestion that post-translational degradation of mutants is a result of partial protein misfolding. But how do SLFN14 mutants mediate SLFN14 WT degradation? All AAA proteins, which SLFN14 belongs to, tend to form oligomers in the cell. Consistently, co-IP and native PAGE revealed that SLFN14 WT form complexes with mutant SLFN14, forming oligomers of different order, and mutations do not affect the protein pattern in the gel. Therefore, SLFN14 mutants could cause increased degradation of wild-type SLFN14 by forming hetero-oligomers of wild-type/mutant SLFN14 leading to instability of the entire protein complex, and could explain a dominant-negative effect of mutant allele on SLFN14 WT expression in patients.

What are the implications of our findings for platelet biogenesis and IT-related dysregulation of this process? It is well known that mature platelet and erythrocytes have only residual “RNA content” levels, if any (Hamilton 2010; Angénieux et al. 2016). Here we show that SLFN14 reveals the endoribonucleolytic activity resulting in rRNA cleavage and degradation in different transfected cells. Therefore, we cautiously speculate that SLFN14 causes RNA degradation and, in such a way, mediates RNA clearance during platelet and erythrocyte maturation. Interestingly, SLFN14 was found abundant in rabbit reticulocytes, but below the detection limit in rabbit liver, lung, and brain tissues (Pisareva et al. 2015). Moreover, endogenous SLFN14 expression was shown to be undetectable or extremely low in HEK293, HEK293T, HeLa, CEM, and Jurkat cells (Li et al. 2012). On the other hand, in the independent studies, SLFN14 was demonstrated to be involved in platelet biogenesis. Taking these data together, we again cautiously hypothesize that SLFN14 is specifically expressed and acts during these two blood cell types maturation processes.

Interestingly a recently published and more global approach (Mills et al. 2016) was studied to outline a ribosomal rescue pathway which involves both erythroid cells and platelets and the many proteins involved in this process such as proteins not dissimilar to SLFN14. Therefore, our more specific study follows on from this and complements this study by implicating a ribosomal pathway more specifically in SLFN14 as outlined here.

Since we did not detect any difference between SLFN14 WT and mutants in our experiments, except at the expression level, we think that the dysregulation of thrombopoiesis is a result of improper degradation of mutants. There are examples of human genetic diseases caused by the degradation of mutant proteins despite these proteins retaining their functionality (Valastyan and Lindquist 2014). A canonical example is the disease cystic fibrosis linked to a single phenylalanine residue deletion at position 508 of cystic fibrosis transmembrane conductance regulator protein targeting a misfolded protein for degradation (Qu et al. 1997). Another case of improper degradation-associated disease is in Gaucher's disease, which is caused by a variety of mutations in β-glucosidase (Futerman and van Meer 2004; Cox and Cachón-González 2012). In conclusion, our findings contribute to the understanding of the mechanism underlying platelet biogenesis in general and SLFN14-related IT more specifically.

## MATERIALS AND METHODS

### Plasmids

Mammalian expression vector containing the full coding region of human *SLFN14* and GFP-tag was purchased from GeneCopoeia [SLFN14(WT)-GFP]. Mammalian expression vector for human SLFN14(WT)-myc as well as *E. coli*-based expression vectors for His-tagged carboxy-terminal deletion mutants of human *SLFN14* [SLFN14(WT)-65 kDa and SLFN14(WT)-45 kDa] have been previously described (Fletcher et al. 2015; Pisareva et al. 2015). The *SLFN14* missense mutations K218E, K219N, and V220D were created by site-directed mutagenesis of corresponding vectors.

### Antibodies

We used myc (Cell Signaling Technology, #9B11), myc (Abcam, #9106), 5.8S rRNA (Abcam, #ab37144), SLFN14 (Abcam, #ab106406), RPS19 (Bethyl Laboratories, #A304-002A), RPL3 (Bethyl Laboratories, #A305-007A), GFP (Sigma, #G1544), GAPDH (Abcam, #ab9485), anti-rabbit AlexoFluor488 (ThermoFisher Scientific, #A-11034), and anti-mouse AlexoFluor568 (ThermoFisher Scientific, #A-11004) antibodies.

### Cell culture, plating, and transfection

HEK293T cells were cultured in DMEM plus l-glutamine (Invitrogen) (plus 10% fetal calf serum, 1% pen/strep [both from GIBCO]). Dami cells were cultured in RPMI (GIBCO) plus 1% l-glutamine, 10% fetal calf serum, and 1% pen/strep. Cells were plated into six-well plates with/without sterilized 23-mm glass coverslips at a density of 5 × 10^5^ cells/mL. Dami medium was supplemented with PMA (Phorbol 12-myristate 13-acetate [Sigma-Aldrich]) to a final concentration of 10 µM. Cells were transfected 24 h post-plating with 5.5 µL (1 mg/mL at pH 7.4 PEI [Polyethyleneimine; Sigma-Aldrich]), 1.2 µg of DNA, and 140 µL of Optimem (Invitrogen) per well of a six-well plate and used in studies 24–48 h post-transfection.

### Purification of ribosomal subunits

Native rabbit 40S and 60S subunits were purified as described (Pisarev et al. 2007).

### Purification of *E. coli*-expressed SLFN14 WT and mutants

Recombinant SLFN14(WT/mut)-45 kDa were expressed in 1 L of *E. coli* BL21(DE3) media after induction with 0.1 mM IPTG for 16 h at 16°C. After expression, the proteins were isolated by affinity chromatography on Ni-NTA agarose followed by FPLC on a MonoS column. FPLC fractions were collected across a 100–500 mM KCl gradient. SLFN14(WT/mut)-45 kDa were eluted in the range of 210–250 mM KCl.

Recombinant SLFN14(WT/K218E)-65 kDa were expressed in 1 L of *E. coli* BL21(DE3) media after induction by 0.1 mM IPTG for 16 h at 16°C and purified on Ni-NTA agarose according to manufacturer's protocol.

### HEK293T cell extract preparation

To prepare cell extract, transiently transfected HEK293T cells expressing SLFN14(WT/mut)-myc or harbouring EV were cultured, plated and transfected as described previously. Prior to use cells were washed three times with 1× PBS (pH 7.4). HEK293T cells (3 × 10^5^ cells) were resuspended in 300 µL of prechilled Tris-based lysis buffer (20 mM Tris-HCl at pH 7.5, 100 mM potassium chloride, 2.5 mM magnesium chloride, 1 mM DTT, 0.1 mg/mL cycloheximide, 0.5% Triton X-100, 40 units/mL DNase I from NEB, HALT protease inhibitor cocktail EDTA-free from Thermo Scientific). The cells were allowed to swell for 10 min on ice and centrifuged at 16,000*g* for 10 min at 4°C.

### Immunoblot analysis and densitometry

Transiently transfected HEK293T cells expressing SLFN14(WT/mut)-myc/myc EV and SLFN14(WT)-GFP were lysed as above and analyzed using densitometry after immunoblotting. Western blot band intensity was quantified in NIS Elements version 4.00.07 as follows: The ROI selection tool was used to draw around the largest band and the average intensity was measured. This box was used to measure the average band intensity of other bands. Background intensity was measured using the same ROI box moved to 4× nonband region in the same lane as the band measured. These values were logged to Excel; for both SLFN14 and GAPDH the average band value was then subtracted from the average background value. To correct for minor differences in protein levels seen in the GAPDH protein control, the band value for SLFN14 was divided by the average band value for GAPDH.

### Coimmunoprecipitation

Cell extract lysates were prepared from transiently transfected HEK293T cells expressing SLFN14(WT)-myc, Myc EV and SLFN14(WT/mut)-GFP or harbouring empty GFP vector as described previously. Protein concentrations in lysates were normalized using Bradford Reagent (Sigma) and absorbance was measured in the 595 nm region using a VersaMax spectrophotometer (Molecular Devices). Protein G Sepharose (PGS) (Sigma) beads were washed three times in PBS and allowed to hydrate in pH 7.4 TBS-T (0.2 M Tris base [Sigma], 1.5 M NaCl [Sigma], 0.05% Tween20 [Sigma[) for 30 min. Lysates were precleared by addition of 25 µL of hydrated PGS beads for 30 min at 4°C, followed by 9000 rpm centrifugation for 15 min at 4°C. The supernatant was removed and 5 µg of anti-myc antibody was added to the lysates alongside 50 µL of hydrated PGS beads and rotated overnight at 4°C. Beads were pelleted at 9000 rpm for 1 min at 4°C. Supernatant was discarded and the beads washed three times in lysis buffer, repelleting in between washes. Fifty microliters of 3× reducing sample buffer was added to the beads and boiled at 105°C for 5 min. Whole-cell and IP lysates were analyzed by Western blot.

### HEK293T ribosomal fractionation

To perform ribosomal fractionation, 100 µL of HEK293T cell extract was diluted with 300 µL of buffer A (20 mM Tris-HCl at pH 7.5, 100 mM KCl, 2.5 mM MgCl_2_, 1 mM DTT, 0.1 mg/mL cycloheximide) and subjected to centrifugation through 10%–50% SDG prepared in buffer B (20 mM Tris-HCl at pH 7.5, 100 mM KCl, 15 mM MgCl_2_, 1 mM DTT, 0.1 mg/mL cycloheximide) in a Beckman SW41 rotor at 35,000 rpm for 150 min at 4°C. After centrifugation, 200-µL fractions were collected. Ribosomal fractions were concentrated and analyzed by immunoblotting.

### Ribosomal binding assay

SLFN14(WT)-65 kDa or SLFN14(K218E)-65 kDa proteins in the form of eluates after Ni-NTA resin were mixed with 50 pmol 80S ribosomes, reconstituted from purified 40S and 60S ribosomal subunits, in the presence of 1 mM AMPPNP. The reaction mixture was subjected to centrifugation through 10%–30% SDG prepared in buffer C (20 mM Tris-HCl at pH 7.5, 100 mM KCl, 2.5 mM MgCl_2_, 1 mM DTT) in a Beckman SW55 rotor at 53,000 rpm for 75 min at 4°C. The 80S ribosomal peak was assayed by NuPAGE 4%–12% Bis-Tris SDS-PAGE (Invitrogen) and SimplyBlue SafeStain (Invitrogen) staining.

### RNA extraction

Total RNA extraction was performed using TRIzol reagent according to the manufacturer's instructions (Life Technologies). Total RNA concentration was determined using a Nanodrop Lite spectrophotometer (Thermo Fisher Scientific).

### Denaturing agarose/formaldehyde gel electrophoresis analysis of rRNA degradation

To study rRNA degradation in HEK293T cell extract, 1 µg of each total RNA sample was analyzed by denaturing agarose/formaldehyde gel electrophoresis. RNA samples were loaded in a loading buffer (5× = 4 mM EDTA, 0.9 M formaldehyde, 20% glycerol, 30.1% formamide, 4× FA buffer, 0.4 µg/mL bromphenol blue) onto 1.2% denaturing agarose/formaldehyde gel (3% formaldehyde) prepared in FA buffer (20 mM MOPS at pH 7.0, 5 mM NaAc, 1 mM EDTA, 0.1 µg/mL ethidium bromide) and were resolved in FA buffer for 22 min at 4°C at 200 V. After electrophoresis, gel was stained twice for 8 min each with 0.5 µg/mL ethidium bromide solution in water, washed three times for 5 min each with water and analyzed using shortwave UV (254 nm).

### RNA integrity analysis on Agilent 2200 TapeStation system

After RNA isolation, total RNA was checked for the integrity of the 18S and 28S rRNAs on the Agilent 2200 TapeStation system (Agilent Technologies). One microgram of each total RNA sample was analyzed. The Agilent software generates an electropherogram profile and gel-like image providing a detailed visual assessment of the quality of an RNA sample. To remove individual interpretation in RNA integrity, the Agilent 2200 TapeStation system also provides information about RNA quality in the form of a RIN. The RIN software algorithm takes into account the entire electrophoretic trace and allows for the classification of eukaryotic total RNA, based on a numbering system from 1 to 10, with 1 being the most degraded profile and 10 being the most intact.

### qRT-PCR

To evaluate the relative *SLFN14* transcript level in cells, 1 µg of isolated total RNA was converted into cDNA using random hexamers and SuperScript III kit (Invitrogen). Ten nanograms of cDNA was subjected to real-time PCR using iQ SYBR Green Supermix kit (Bio-Rad) with primers

SLFN14 qPCR dir 5′-GCAAAGAAGTGGTTGGATGTAAG-3′,
SLFN14 qPCR rev 5′-TCACAGCAGAAGTGGAATGTAG-3′,
GAPDH qPCR dir 5′-GGTGTGAACCATGAGAAGTATGA-3′
GAPDH qPCR rev 5′-GAGTCCTTCCACGATACCAAAG-3′,

and CFX96 Touch Real-Time PCR Detection System (Bio-Rad). *SLFN14* transcription level was normalized to GAPDH control.

### SLFN14 oligomerization assay

Five micrograms of SLFN14(WT/mut)-45 kDa were analyzed by nondenaturing NativePAGE 4%–16% Bis-Tris PAGE (Invitrogen) and SimplyBlue SafeStain (Invitrogen) staining in the presence of NativeMark Unstained Protein Standard (Invitrogen).

### Steady-state fluorescence measurements

Fluorescence emission spectra were obtained on a Fluoromax-3 spectrophotometer (Jobin Yvon Inc., Edison, NJ). Six micrograms of SLFN14(WT)-45 kDa or SLFN14(K218E)-45 kDa in 200µL of buffer C were incubated at 25°C or 65°C for 10 min. Protein fluorescence was monitored using an excitation wavelength of 280 nm and an emission wavelength range of 300–400 nm.

### Immunocytochemistry

Dami cells (American Type Culture Collection) were plated onto glass coverslips and transfected with SLFN14(WT/mutant)-myc as described above. Cells were fixed in 4% PFA for 5 min, and permeabilized in 0.1% Triton-X-100 in PBS for 5 min. Cells were incubated in block buffer (PBS [Invitrogen], 10% goat serum [Gibco], 5% BSA) for 1 h. Cells were then incubated for 1 h in primary antibody diluted in block as per manufacturer's instructions. Cells were washed in PBS and incubated for 1 h in secondary antibody plus Topro-3 (Invitrogen) diluted as per manufacturer's instructions in block. Cells were mounted on glass slides using Hydromount mounting media (National Diagnostics).

### Immunofluorescence microscopy and analysis

All images were taken using a DM IRE2 Leica inverted microscope, SP2 confocal system running Leica Confocal Software Version 2.61 Build 1537. Confocal imaging was performed using the 488 nm line of an Argon-Ion laser 457–514 nM (to image AlexaFluor488 labeled constructs) and the 568 and 633 line of the HeNe lasers (to image AlexoFluor558 labeled constructs and TOPRO-3) with an HCX Plan Apo Ibd.BL 63× NA 1.4, Olympus objective. *Z*-stack images were taken at 10 slices per cell. Images were analyzed using NIS Elements Software.

Colocalization between SLFN14(WT/Mut)-myc and 5.8S rRNA was performed as follows: From average intensity projections the automated ROI tool in NIS-Elements, the entire cell volume, the cell cytoplasm and the nucleus of each cells were selected. Subsequently, a Pearson's coefficient comparing SLFN14(WT/Mut)-myc and 5.8S rRNA signals in these regions were obtained. In order to control for random colocalization, the 5.8S RNA image was rotated by 5°C and a Pearson's coefficient repeated. All values were logged to Excel (Microsoft) A Student's Test was performed to ascertain statistical significance between colocalization within the various subcellular regions for wild-type of mutant SLFN14 and as a control between the rotated and nonrotated images.

#### Intensity measurements

Average intensity projections were created and ROIs drawn around the entire cell volume of a bright-field image of both nontransfected cells and cells expressing SLFN14(WT/Mut)-myc images within the same field of view. This ROI outline was superimposed over the 5.8S rRNA stained image and the average intensity measurement within the ROI calculated. All values were logged to Excel (Microsoft). A Student's test was performed to ascertain statistical significance between 5.8S RNA staining intensity between transfected and nontransfected cells.
